# A Multidimensional Virtual Reality Neurorehabilitation Approach to Improve Functional Memory: Who Is the Ideal Candidate?

**DOI:** 10.3389/fneur.2020.618330

**Published:** 2021-01-14

**Authors:** Sonia Di Tella, Sara Isernia, Chiara Pagliari, Johanna Jonsdottir, Carlotta Castiglioni, Patrizia Gindri, Cristina Gramigna, Samuela Canobbio, Marco Salza, Franco Molteni, Francesca Baglio

**Affiliations:** ^1^IRCCS Fondazione Don Carlo Gnocchi ONLUS, Milan, Italy; ^2^Fondazione Opera San Camillo Presidio Sanitario San Camillo, Turin, Italy; ^3^Villa Beretta Rehabilitation Center, Ospedale Valduce, Como, Italy

**Keywords:** rehabilitation, telerehabilitation, virtual reality, multiple sclerosis, stroke, Parkinson disease, digital health, cognition

## Abstract

**Aims:** We aimed to identify the significant predictors of ecological memory amelioration after the Human Empowerment Aging and Disability (HEAD) rehabilitation program, a multidimensional treatment for chronic neurological diseases.

**Materials and Methods:** Ninety-three patients with Parkinson disease (*n* = 29), multiple sclerosis (*n* = 26), and stroke (*n* = 38) underwent a multidimensional rehabilitation. We focused on changes after treatment on ecological memory (outcome measure) evaluated by Rivermead Behavioral Memory Test, Third Edition (RBMT-3). Minimal clinically important difference (MCID) after treatment were calculated for RBMT-3. The change score on RBMT-3 was categorized in positive effect, stabilization, or no effect of the treatment. Random forest classification identified who significantly benefited from treatment against who did not in terms of ecological memory functioning. Accordingly, logistic regression models were created to identify the best predictors of the treatment effect. A predicted probability value was derived, and the profile of the ideal candidate of HEAD protocol was shown by combining different ranks of significant predictors in a 3 × 3 matrix for each pair of predictors.

**Results:** A significant number of cases reported positive effect of the treatment on ecological memory, with an amelioration over the MCID or a stabilization. The random forest analysis highlighted a discrete accuracy of prediction (>0.60) for all the variables considered at baseline for identifying participants who significantly benefited and who did not from the treatment. Significant logistic regression model (Wald method) showed a predictive role of Montreal Cognitive Assessment (MoCA; *p* = 0.007), 2**-**Minute Walk Test (2MWT; *p* = 0.038), and RBMT-3 (*p* < 0.001) at baseline on HEAD treatment effect. Finally, we observed a high probability of success in people with higher residual cognitive functioning (MoCA; odds ratio = 1.306) or functional mobility (2MWT; odds ratio = 1.013).

**Discussion:** The HEAD program is a rehabilitation with effects on multiple domains, including ecological memory. Residual level of cognitive and/or motor functioning is a significant predictor of the treatment success. These findings confirm the intrinsic relationship subsisting between motor and cognitive functions and suggest the beneficial effects of physical activity on cognitive functions and *vice versa*.

## Introduction

Recent reports alarmingly pointed out the age-related increment of years of life with diseases (1). Since 1990, mortality rates declined concomitantly with the growth of non-fatal diseases, leading people to cope with chronic conditions and consequently chronic care needs throughout life. Parkinson disease (PD), multiple sclerosis (MS), and post-stroke are the most prevalent chronic neurological conditions (2–4) that weigh heavily on the personal burden and the healthcare costs (5). Especially, Global Burden of Diseases' studies recently reported a global prevalence of more than 6 million PD cases (1), more than two million of MS patients (6), and about 1 million adults living with stroke (2). Although these conditions are characterized by different epidemiology and etiopathology, they are united by a high level of motor and cognitive disability accounting for a consistent loss of quality of life. Regarding the cognitive profile, cognitive deficits are heterogeneous, but memory and executive dysfunctions are frequently reported in all of them (7–9). It is of great importance to cope with the cognitive deficits considering their significant impact on daily living (10). Specifically, everyday memory difficulties are frequent and common in MS, PD, and stroke diseases (11–13). Intact memory skills are required to complete many everyday activities; thus, impairments in memory functioning can have important negative effects on the individual ability to live independently and negative implications for quality of life. Given the chronic course of the disease, people living with these conditions must cope with disability for the remainder of their lives. For this reason, new rehabilitative solutions for such individuals to preserve or improve cognitive status and everyday functioning are crucial; especially, it is important to evaluate their efficacy adopting an ecological assessment. Recent evidence suggests (1) the extensive beneficial effects of multidimensional rather than unidimensional treatment, (2) the positive results from the integration of virtual reality (VR) systems into the conventional rehabilitation in people with chronic neurological diseases, and (3) the importance of characterizing the profile of the ideal candidate for these novel approaches.

First, because of the multidimensional pathology-related difficulties, often impacting motor, cognitive, and behavioral functionality, multidisciplinary models of care are taken in consideration (14–17). Recently proposed integrated treatments involve a multidisciplinary team to offer a personalized systemic care for the disabled person. This holistic approach provides beneficial effects in everyday living, and, for this reason, tools to detect changes in daily functioning after treatments need to be considered. In fact, in the last few years, it has become increasingly clear that standard paper-and-pencil neuropsychological tests are limited in predicting what occurs in patients' everyday life. Only weak associations were reported between results on classical tests and subjects' complaints of everyday problems (18–21). To overcome these difficulties and to better describe how cognitive deficits may affect daily functioning, an innovative approach has been proposed, which entails the administration of more ecological tasks (22, 23).

The second evidence regards the adoption of VR solutions. Rehabilitation with these tools seems to be promising in terms of patient involvement and treatment efficacy (24, 25). The utilization of these VR tools helps facilitate engagement and increase patient satisfaction during the training (26–31), by creating a virtual environment eliciting realistic perceptions and reactions (32). In this framework, the Human Empowerment Aging and Disability (HEAD) protocol is a VR multidimensional rehabilitation intervention for people with chronic neurological conditions conceived for both clinical and home settings (i.e. telerehabilitation). A previous study demonstrated its feasibility (33) and its efficacy in PD populations (24). However, as an integrated treatment proposed for different pathologies and grades of disabilities, a secondary investigation on the predictors of treatment success on everyday functions can provide extensive information on the population target for the HEAD rehabilitation.

The last consideration focused on the lack of clinical consensus regarding the characteristics of the population for targeting these types of VR treatments. It is extremely useful to identify which clinical features are prognostic of treatment success. Along these lines, a new field of investigation aims to individualize significant predictors of treatments (34, 35). This approach establishes the profile of the ideal candidate for a given rehabilitation intervention. This strategy will facilitate the possibility to *a priori* differentiate between patients who will potentially benefit from the treatment and those who will not. The implications of these studies are large and favor the personalization of intervention targeted for the patient, by ensuring a high probability of treatment success.

The present study aims to characterize the profile of the ideal candidate for the VR-multidimensional treatment who will benefit the most with a high probability on ecological measures. Accordingly, we performed a secondary analysis on a large cohort of patients who completed a VR multidimensional treatment (the HEAD program) by adopting an ecological measure of cognitive functioning, one of the more disabling aspects of the chronic neuropathological conditions.

## Methods

This study consists of a secondary analysis on data related to a multicenter interventional protocol of integrated rehabilitation for people with chronic neurological diseases whose efficiency and efficacy findings are described elsewhere (24, 33). In this context, we focus on the first part of the study design in which patients underwent a 1-month rehabilitation period in and outpatient setting, consisting in 45-min sessions three times per week, for a total of 12 sessions (ClinicHEAD). The entire dataset from the three recruiting centers of the original study (Valduce Hospital Villa Beretta Rehabilitation Center in Lecco, IRCCS Don Carlo Gnocchi Foundation in Milan and the District Clinic San Camillo in Turin) was utilized for the present work. The study was carried out under the norms of the Declaration of Helsinki; it was approved by the local ethics committees; each participant was adequately informed about the study and offered their collaboration and signed a written informed consent.

### Participants

The sample of the present study consists of people with chronic neurological conditions meeting the following inclusion criteria: diagnosis of MS with an Expanded Disability Status Scale score ≤ 5.5, or diagnosis of PD with a Hoehn and Yahr score ≤ 2, or diagnosis of chronic stroke at least 6 months after the event; ages between 18 and 80 years; Mini-Mental State Examination score >20; absence of disabling pain; severe deficit of visual acuity or auditory perception or in communication; and absence of severe dysmetria.

Patients were enrolled during their periodical clinical visit by the neurologists, periodically receiving neurological follow-up.

All subjects took part in an experimental clinical trial between 2016 and 2017 consisting of multidimensional rehabilitation with VR activities in the clinic, lasting 1 month, 3 times a week for 12 sessions. The intervention, extensively detailed elsewhere (33), took place in the clinic, with the presence of clinical professionals: the neurologist, the physiotherapist, and the neuropsychologist. Motor and cognitive rehabilitation activities were proposed while interacting with virtual scenarios and watching short video clips. The rehabilitation dimensions targeted by the treatment included balance, endurance, speed and strength of both upper and lower limbs, executive functions, memory, language, and dual-task capabilities.

### Measurements for the Analysis

Cognitive performance outcome was obtained by the Montreal Cognitive Assessment [MoCA; (36)] and the Rivermead Behavioral Memory Test, Third Edition [RBMT-3; (37)]. The MoCA (36), is a sensitive tool for global cognitive level assessment, by screening different domains, such as executive functions, memory, language, visual–spatial abilities, attention, calculation, abstraction, and spatial and temporal orientation (scores range from 0 to 30). Two parallel forms of this instrument (38) were utilized for the assessment at T0 and T1. Following Santangelo et al. (39) scores correction procedure, we obtained a age and education adjusted score of the MoCA subdomains: visuospatial abilities (AVS), executive functions (EF), memory (ME), attention (ATT), language (LANG), and orientation (OR).

RBMT-3 is an ecological battery for the assessment of everyday memory performance with relatively short times of administration, and parallel forms and is applicable to patients with motor deficits (37). The RBMT-3 consists of 14 subtests (scores range from 51 to 147): names (remembering the first and second names of two portrait photos), belongings (remembering to ask for two personal belongings at the end of the evaluation session), appointments (asking two questions when an alarm rings 25 min later), picture recognition (delayed recognition of line drawings against distractors), story (immediate and delayed recall of a short story), faces (delayed recognition of photographs of faces against distractors), route (immediate and delayed recall of a short route in the examination room), message (immediate and delayed remembering to pick up an envelope and book), orientation and date (orientation to person, place and time), and novel task (immediate and delayed recall of puzzle pieces positioned in a specific order within a template). In addition to the scaled scores on the subtests, the Global Memory Index (GMI) was calculated as an overall memory performance measure.

Motor performance outcome was evaluated by the Berg Balance Scale [BBS; (40)], 10-Meter Walk Test [10MWT; (41)], and 2-Minute Walk Test [2MWT; (42)]. The BBS is a measure of static balance and the risk of falling. It consists of a 14-item 4-point scale, with a total score ranging from 0 to 56. 10MWT is a quantitative analysis of the walking speed, measuring the speed in meters per second over 10 m. It is considered an assessment of functional mobility. The 2MWT provides a quantitative analysis of gait speed and endurance. The walking distance walked in 2 min is registered as a functional mobility measure.

Measures of quality of life and affectivity were also considered: the Positive Affect and Negative Affect Schedule [PANAS; (43)]. The PANAS scale consists of 20 items that evaluate two independent dimensions: positive affect and negative affect. The range for each scale (10 items on each) is from 10 to 50.

### Statistical Analyses

Statistical analyses on outcome measures were performed with IBM SPSS Statistics software (version 24) and JASP (JASP Team 2020, JASP version 0.11.1).

Means, frequencies, and standard deviations were computed to describe sample characteristics. χ^2^-test and univariate analysis of variance were used to verify whether the three pathologies included in the sample were balanced for age, education, and sex distribution.

For each outcome measure, changes scores (Δ change) from T1 to T0 were calculated. Minimal Clinical Important Difference (MCID) was derived separately for each pathology computing one-half of the deviation standard, according to Katajapuu et al. (44) and Shikiar et al. (45). After that, each change score was categorized into one of three categories: positive effect of the treatment (Δ change > MCID), stable after treatment (–MCID ≤ Δ change ≤ MCID), and no effect of the treatment (Δ change < MCID). Frequencies and χ^2^-test were run to show effectiveness results of the treatment on the whole sample and separately for each pathology.

Random forest (RF) classification was applied to the data as an exploratory analysis including all demographic and clinical variables assessed at the baseline, as an overall prediction approach in identifying subjects who significantly benefited from treatment in the RBMT-3 (Δ change > MCID). For this purpose, the RBMT-3 outcome was considered dichotomously (Δ change > MCID vs. Δ change ≤ MCID). We built RFs with the default parameter values in JASP (version 0.11.1), with the exception of the data split for which we partitioned the data set into a training (50%), validation (20%), and test set (30%). In relation to the number of trees, we selected an optimal number of trees [Ntrees (maximum) = 100], optimized with respect to the out-of-bag accuracy. Classification accuracy represents the proportion of the instances that were classified correctly.

Performance of the classification model was also evaluated by carrying out a receiver operating characteristic (ROC) analysis. The area under the ROC curve (AUC) provides a measure of overall prediction accuracy and corresponds to random chance when AUC is equal to 0.5 and represents perfect accuracy when AUC be 1. Precision represents the proportion of true positives among all the instances classified as positive; F1 score indicates the harmonic mean of precision and recall, and recall is the proportion of cases that were classified as positive, among all instances that truly were positives.

To further explore the link between the dichotomic variable of the outcome RBMT-3 and possible predictors at baseline, point-biserial correlation analyses were performed for continuous variables and χ^2^-test for categorical variables to select variables for insertion in the following regression model. A *p* < 0.10 was preferred to the conventional threshold *p* < 0.05 to avoid excluding potential significant predictors.

A logistic regression model was utilized to identify the best predictors of treatment effect. Regardless the statistical significance of association in the previous phase (χ^2^-test), the pathology (PD, SM, stroke) was cautiously considered in the regression model as a possible predictor. Wald forward option was used as a stepwise selection method. A predicted probability value was derived from the logistic model for each subject. Finally, significant baseline predictors were organized in three-tile ranks, and the mean predicted probability values were shown by combining the different ranks of predictors (in a 3 × 3 matrix for each pair of significant predictors).

## Results

### Participants

Ninety-three of the 112 subjects of the original dataset were considered for the present study as they had no missing data (29 with PD, 26 with MS, and 38 with a stroke in the chronic phase). The three pathologies were balanced in terms of sex distribution and level of education. The age of the MS group significantly differed from PD and stroke (Table 1).

**Table 1:** Description of sample characteristics at baseline.

	**PD**	**MS**	**Stroke**	**All**	**Groups** **comparison *p*-value**	**Pairwise** **comparisons**
*n*	29	26	38	93	—	
Sex (Ma:F)	15:14	13:13	21:17	49:44	0.911^∧^	
Age (mean ± s.d.)	66.21 ± 9.09	50.96 ± 11.41	59.66 ± 12.29	59.27 ± 12.49	**<0.001**^**§**^	MS < PD/stroke
Education (mean ± s.d.)	11.86 ± 4.42	11.50 ± 3.20	12.89 ± 3.97	12.18 ± 3.93	0.347^#^	
2MWT (mean ± s.d.)	133.17 ± 35.77	95.25 ± 37.67	78.49 ± 44.88	100.23 ± 46.14	**<0.001**^**§**^	MS/stroke < PD
MMSE (mean ± s.d.)	27.52 ± 1.92	27.27 ± 2.11	26.84 ± 2.84	27.17 ± 2.38	0.506^∧^	
MoCA (mean ± s.d.)	22.33 ± 2.65	20.08 ± 3.38	20.02 ± 4.29	20.76 ± 3.71	**0.021**^**§**^	PD > stroke
RBMT-3–GMI (mean ± s.d.)	85.07 ± 17.89	60.73 ± 14.61	80.66 ± 17.29	78.70 ± 17.84	**0.002**^****§^	MS < PD/stroke
BBS (mean ± s.d.)	48.93 ± 6.39	42.81 ± 9.98	40.13 ± 15.36	43.62 ± 12.19	**0.018**^#^	MS/stroke < PD
10MWT (mean ± s.d.)	6.94 ± 4.97	8.51 ± 4.10	15.12 ± 12.02	10.72 ± 9.17	**<0.001**^**#**^	stroke>PD/MS
PANAS-PA (mean ± s.d.)	33.52 ± 8.21	34.69 ± 6.10	35.34 ± 7.84	34.59 ± 7.48	0.529^#^	
PANAS-NA (mean ± s.d.)	17.90 ± 7.02	17.08 ± 7.23	15.95 ± 7.65	16.87 ± 7.31	0.343^#^	

2MWT, 2-Minute Walk Test; 10MWT, 10-Meter Walk Test; BBS, Berg Balance Scale; F, females; M, mean; Ma, males; MMSE, Mini Mental State Examination; MoCA, Montreal Cognitive Assessment; MS, multiple sclerosis; N, number; PANAS-PA, Positive Affect and Negative Affect Schedule—positive affect; PANAS-NA, Positive Affect and Negative Affect Schedule—negative affect; PD, Parkinson disease; RBMT-3–GMI, Rivermead Behavioral Memory Test, Third Edition—Global Memory Index; s.d., standard deviation.

∧ χ^2^-test computed;

§ univariate analysis of variance computed;

# *Kruskal–Wallis test computed. p < 0.05 are reported in bold*.

The global cognitive level at the Mini-Mental State Examination was comparable between the three groups. However, when considering the MoCA total score, patients with PD showed higher global cognitive functioning than stroke. Moreover, the memory profile at RBMT-3–GMI was slightly different between MS and the other two conditions (MS < PD/stroke). The specific profile on MoCA and RBMT-3 subscores is detailed in Table 2.

**Table 2:** Description of sample cognitive profile at baseline.

	**PD**	**MS**	**Stroke**	**All**	**Groups** **comparison** ***p* value**	**Pairwise** **comparisons**
n	29	26	38	93	—	
MoCA subscore [median (IQR)]						
AVS	2.96 (1.46)	3.65 (1.52)	3.15 (1.70)	3.23 (1.58)	0.738	
EF	2.78 (1.96)	2.44 (2.63)	2.19 (1.73)	2.57 (2.37)	0.331	
ME	1.00 (3.50)	2.00 (3.00)	2.00 (3.25)	2.00 (3.00)	0.875	
ATT	6.00 (0.68)	6.00 (1.16)	5.87 (1.60)	6.00 (1.12)	0.237	
LANG	5.42 (1.55)	5.03 (1.65)	4.65 (2.08)	4.90 (1.75)	0.072	
OR	6.00 (0.00)	6.00 (0.00)	6.00 (0.95)	6.00 (0.00)	0.381	
RBMT subscores [median (IQR)]						
N	8.00 (6.00)	5.50 (4.00)	6.00 (4.50)	6.00 (6.00)	**0.016**	MS < PD
B	9.00 (7.50)	11.00 (4.25)	11.00 (5.50)	11.00 (7.00)	0.828	
A	8.00 (6.50)	5.50 (6.25)	8.50 (8.00)	8.00 (7.00)	**0.015**	MS < PD/stroke
PR	12.00 (2.00)	12.00 (4.00)	12.00 (3.00)	12.00 (3.00)	0.475	
SI	7.00 (3.00)	5.00 (5.25)	7.00 (6.00)	7.00 (4.50)	0.112	
SD	6.00 (3.00)	4.00 (4.00)	6.00 (4.25)	5.00 (3.00)	0.050	
FR	11.00 (4.50)	7.00 (5.00)	9.00 (5.00)	9.00 (6.00)	**0.005**	MS < PD
RI	10.00 (6.00)	7.50 (6.50)	10.00 (6.00)	9.00 (7.00)	**0.012**	MS < PD/stroke
RD	9.00 (6.50)	6.50 (7.25)	8.00 (7.50)	8.00 (7.50)	0.088	
MI	11.00 (4.00)	6.50 (10.00)	11.00 (6.50)	11.00 (7.00)	**0.022**	MS < PD/stroke
MD	12.00 (4.00)	11.00 (10.25)	11.50 (7.00)	11.00 (7.00)	0.060	
O	8.00 (5.50)	7.50 (5.00)	9.00 (4.50)	9.00 (4.00)	0.110	
NI	6.00 (5.50)	2.00 (5.00)	4.00 (6.00)	5.00 (6.00)	**0.004**	MS < PD
ND	6.00 (2.50)	1.00 (2.00)	4.00 (5.00)	4.00 (4.50)	**0.001**	MS < PD/stroke

*Differences between the three groups were tested with Kruskal–Wallis test. PD, Parkinson disease; MS, multiple sclerosis; MoCA, Montreal Cognitive Assessment; AVS, visuospatial abilities; EF, executive functions; ME, memory; ATT, attention; LANG, language; OR, orientation; RBMT-3, Rivermead Behavioral Memory Test, Third Edition; IQR, interquartile range; N, Names–Delayed Recall; B, Belongings–Delayed Recall; A, Appointments–Delayed Recall; PR, Picture Recognition; SI, Story–Immediate Recall; SD, Story–Delayed Recall; FR, Face Recognition–Delayed Recall; RI, Route–Immediate Recall; RD, Route–Delayed Recall; MI, Messages–Immediate Recall; MD, Messages–Delayed Recall; O, Orientation and Date; NI, Novel Task–Immediate Recall; ND, Novel Task–Delayed Recall. p < 0.05 are reported in bold*.

The three groups showed an equal level of affectivity, whereas a major impairment in motor functioning was observed in stroke (Table 1).

### Treatment Effects

Changes between T1 and T0 were classified in one of three categories: patients who significantly benefited from treatment (Δ change > MCID), patients who substantially remained stable after treatment (–MCID ≤ Δ change ≤ MCID), and patients with a significant worsening over time (Δ change < MCID).

Percentages of treatment success for each outcome measure are reported in Table 3. The results showed a significantly higher number of cases with treatment success and who remained stable after treatment vs. patients with a significant worsening over time in all outcomes related to cognitive and motor functioning, and affectivity. Table 4 reports percentages of treatment success separately for each pathology.

**Table 3:** Changes between T0 and T1 and comparison results of treatment effect vs. no effect cases.

		**Δ change (mean ± SD)**	**%** **no treatment success**	**%** **stable after treatment**	**%** **treatment success**	**χ^2^** **(*df* = 2)**	**% success *p* value**
Cognitive functioning	MoCA	1.25 ± 2.43	8.60	51.60	39.78	27.548	**<0.001**
	AVS	0.13 ± 1.04	20.43	46.24	33.33	9.290	**0.010**
	EF	0.63 ± 1.31	12.90	38.71	48.39	18.774	**<0.001**
	ME	0.57 ± 1.48	20.43	31.18	48.39	11.097	**0.004**
	ATT	0.03 ± 1.02	18.28	64.52	17.20	40.710	**<0.001**
	LANG	0.02 ± 1.10	29.03	44.09	26.88	4.903	**0.086**
	OR	0.01 ± 0.77	6.45	81.72	11.83	98.387	**<0.001**
	RBMT-3–GMI	5.94 ± 10.85	9.68	50.54	39.78	25.032	**<0.001**
	RBMT-3 subtests						
	N	1.32 ± 3.55	18.28	40.86	40.86	9.484	**<0.009**
	B	0.75 ± 4.29	15.05	52.69	32.26	19.806	**<0.001**
	A	0.92 ± 3.73	15.05	51.61	33.33	18.645	**<0.001**
	PR	−0.40 ± 3.44	23.66	62.37	13.98	36.581	**0.002**
	SI	1.31 ± 2.98	17.20	29.03	53.76	19.419	**<0.001**
	SD	1.20 ± 2.87	15.05	38.71	46.24	14.774	**0.001**
	FR	−0.76 ± 3.63	34.41	40.86	24.73	3.677	**0.159**
	RI	−0.20 ± 4.41	29.03	45.16	25.81	6.000	**0.050**
	RD	0.40 ± 4.26	23.66	49.46	26.88	11.032	**0.004**
	MI	−0.60 ± 5.21	31.18	48.39	20.43	11.097	**0.004**
	MD	0.04 ± 4.91	21.51	56.99	21.51	23.419	**<0.001**
	O	1.48 ± 3.01	10.75	49.46	39.78	22.645	**<0.001**
	NI	1.97 ± 3.99	19.35	22.58	58.06	25.742	**<0.001**
	ND	1.77 ± 4.16	20.43	36.56	43.01	7.548	**0.023**
Motor functions	2MWT	6.90 ± 19.38	7.53	69.89	22.58	59.097	**<0.001**
	10MWT	−0.86 ± 3.04	1.08	89.25	9.68	131.871	**<0.001**
	BBS	1.61 ± 4.53	3.23	84.95	11.83	112.516	**<0.001**
Affectivity	PANAS-PA	0.20 ± 7.31	29.03	43.01	27.96	3.935	0.140
	PANAS-NA	−2.06 ± 7.02	10.75	58.06	31.18	31.419	**<0.001**

*%, percentage; Δ, delta change between T0 and T1; 2MWT, 2**-**Minute Walk Test; 10MWT, 10-Meter Walk Test; BBS, Berg Balance Scale; M, mean; PANAS-PA, Positive Affect and Negative Affect Schedule—positive affect; PANAS-NA, Positive Affect and Negative Affect Schedule—negative affect; RBMT-3–GMI, Rivermead Behavioral Memory Test, Third Edition—Global Memory Index; SD, standard deviation. N, Names–Delayed Recall; B, Belongings–Delayed Recall; A, Appointments–Delayed Recall; PR, Picture Recognition; SI, Story–Immediate Recall; SD, Story–Delayed Recall; FR, Face Recognition–Delayed Recall; RI, Route–Immediate Recall; RD, Route–Delayed Recall; MI, Messages–Immediate Recall; MD, Messages–Delayed Recall; O, Orientation and Date; NI, Novel Task–Immediate Recall; ND, Novel Task–Delayed Recall. p < 0.05 are reported in bold*.

**Table 4:** Changes within each pathology between T0 and T1 and comparison results of treatment effect vs. no effect cases.

	**PD**			**MS**			**Stroke**				
	**%** **no treatment success**	**%** **stable after treatment**	**%** **treatment success**	**%** **no treatment success**	**%** **stable after treatment**	**%** **treatment success**	**%** **no treatment success**	**%** **stable after treatment**	**%** **treatment success**	**χ^**2**^**	***p***
MoCA	17.2	41.4	41.4	7.7	50.0	42.3	2.6	60.5	36.8	5.445	0.245
AVS	24.1	48.3	27.6	23.1	50.0	26.9	15.8	42.1	42.1	2.416	0.660
EF	6.9	44.8	48.3	11.5	34.6	53.8	18.4	36.8	44.7	2.437	0.656
ME	17.2	41.4	41.4	23.1	30.8	46.2	21.0	23.7	55.3	2.587	0.629
ATT	20.7	65.5	15.8	15.4	65.4	19.2	18.4	63.2	18.4	0.521	0.971
LANG	31.0	41.4	27.6	38.5	46.2	15.4	21.1	44.7	34.2	3.790	0.435
OR	6.9	82.8	10.3	0.0	84.6	15.4	10.5	79.0	10.5	3.117	0.538
RBMT-3–GMI	13.8	51.7	34.5	0.0	46.2	53.8	13.2	52.6	34.2	5.433	0.246
RBMT-3 subtests											
N	17.2	44.8	37.9	11.5	38.5	50.0	23.7	39.5	36.8	2.179	0.703
B	24.1	34.5	41.4	11.5	65.4	23.1	10.5	57.9	31.6	6.569	0.161
A	13.8	41.4	44.8	11.5	38.5	50.0	18.4	68.4	13.2	12.026	**0.017**
PR	37.9	55.2	6.9	19.2	69.2	11.5	15.8	63.2	21.1	6.660	0.155
SI	10.3	17.2	72.4	15.4	38.5	46.2	23.7	31.6	44.7	6.773	0.148
SD	10.3	31.1	58.6	11.5	57.7	30.8	21.0	31.6	47.4	7.228	0.124
FR	37.9	48.3	13.8	30.8	34.6	34.6	34.2	39.5	26.3	3.330	0.504
RI	34.5	41.4	24.1	23.1	38.5	38.5	28.9	52.6	18.4	3.882	0.422
RD	34.5	31.0	34.5	15.4	57.7	26.9	21.0	57.9	21.1	6.295	0.178
MI	27.6	48.3	24.1	15.4	61.5	23.1	44.7	39.5	15.8	6.678	0.154
MD	13.8	62.1	24.1	11.5	61.5	27.0	34.2	50.0	15.8	6.432	0.169
O	13.8	44.8	41.4	3.8	53.8	42.3	13.2	50.0	36.8	1.979	0.740
NI	20.7	13.8	65.5	19.2	30.8	50.0	18.4	23.7	57.9	2.397	0.663
ND	20.7	34.5	44.8	11.5	53.8	34.7	26.3	26.3	47.4	5.518	0.238

*%, percentage; Δ, delta change between T0 and T1; RBMT-3–GMI, Rivermead Behavioral Memory Test, Third Edition—Global Memory Index; SD, standard deviation; N, Names–Delayed Recall; B, Belongings–Delayed Recall; A, Appointments–Delayed Recall; PR, Picture Recognition; SI, Story–Immediate Recall; SD, Story–Delayed Recall; FR, Face Recognition–Delayed Recall; RI, Route–Immediate Recall; RD, Route–Delayed Recall; MI, Messages–Immediate Recall; MD, Messages–Delayed Recall; O, Orientation and Date; NI, Novel Task–Immediate Recall; ND, Novel Task–Delayed Recall. p < 0.05 are reported in bold*.

The RF analyses revealed an overall good accuracy (77.8 %) of the classification model built to identify subjects who significantly benefited from treatment in the RBMT-3 (Δ change > MCID vs. Δ change ≤ MCID). Table 5 shows the predictive performances of RF in terms of Precision, Recall, F1 Score and AUC. Precision was above 60% for both classes of patients who benefited and did not benefit from treatment.

**Table 5:** Evaluation Metrics of RF classification.

**Evaluation metrics**				
	**Precision**	**Recall**	**F1 Score**	**AUC**
Patients who did not benefit from treatment	0.938	0.750	0.833	0.682
Patients who benefited from treatment	0.545	0.857	0.667	0.746
Average/Total	0.836	0.778	0.790	0.714

rea Under Curve (AUC) is calculated for every class against all other classes.

### Possible Predictors of Treatment

By adopting an explorative approach, point-biserial correlations (*rpb*) and χ^2^-test, as appropriate, were run between the dichotomic variable of the outcome RBMT-3–GMI (Δ change > MCID vs. Δ change ≤ MCID) and clinical and demographic data in order to detect potential predictors at baseline.

Results highlighted a link between Δ RBMT-3–GMI (Δ change > MCID vs. Δ change ≤ MCID) and MoCA at baseline (*rpb* = 0.178, *p* = 0.087), visuospatial subdomain (AVS) of MoCA at baseline (*rpb* = 0.211, *p* = 0.042), attention subdomain (ATT) of MoCA at baseline (*rpb* = 0.210, *p* = 0.043), RBMT-3–GMI at baseline (*rpb* = −0.250, *p* = 0.016) (2MWT (*r* = 0.274, *p* = 0.008), BBS at baseline (*r* = 0.196, *p* = 0.060), and 10MWT at baseline (*r* = −0.264, *p* = 0.011).

### Regression Models for the Identification of the Best Predictors of the Treatment

Two logistic regression models were computed considering significant results of correlations and the pathology (PD, SM, stroke) as possible predictors of the outcome RBMT-3–GMI (Δ change > MCID vs. Δ change ≤ MCID). In the first model, the following variables were included in the logistic regression: 2MWT, RBMT-3–GMI, MoCA, BBS, and 10MWT. Instead, in the second model, the MoCA was substituted by the subdomains that resulted significantly associated to RBMT-3–GMI Δ change in the preliminary correlation analysis: AVS and ATT. With respect to the first regression, the final third step (Cox and Snell *R*^2^ = 0.247, Nagelkerke *R*^2^ = 0.334) correctly classified 73.12% of patients. Variables excluded from the third final step were BBS and 10MWT scores at baseline. The binary logistic regression revealed a significant link between RBMT-3–GMI change after rehabilitation and outcome measure at baseline, which was confirmed with a predictive effect for the RBMT-3–GMI, MoCA and 2MWT scores. β-value indicated an inverse relation between the outcome RBMT-3–GMI (Δ change) and the RBMT-3–GMI at baseline, whereas a direct relation was observed between the outcome RBMT-3–GMI (Δ change) and the MoCA and 2MWT scores at baseline the variables (see Table 6 for details).

**Table 6:** Binary logistic regression model to test best predictors of the RBMT-3–GMI change after rehabilitation.

		**β**	**SE**	**Wald**	***p*-value**	**Odds ratio** **(*B*)**
Step 1	2MWT T0	0.013	0.005	6.582	**0.010**	1.013
	Constant	−1.747	0.576	9.187	0.002	0.174
Step 2	2MWTT0	0.018	0.006	9.932	**0.002**	1.019
	RBMT-3–GMI T0	−0.046	0.015	8.844	**0.003**	0.955
	Constant	1.216	1.127	1.164	0.281	3.374
Step 3	2MWT T0	0.013	0.006	4.316	**0.038**	1.013
	MoCA T0	0.267	0.099	7.328	**0.007**	1.306
	RBMT-3–GMI T0	−0.075	0.020	13.921	**<0.001**	0.928
	Constant	−1.510	1.607	0.883	0.347	0.221

*2MWT, 2-Minute Walk Test; MoCA, Montreal Cognitive Assessment; RBMT-3–GMI, Global Memory Index; SE, standard error. p < 0.05 are reported in bold*.

In the second regression, the final third step (Cox and Snell *R*^2^ = 0.235, Nagelkerke *R*^2^ = 0.318) correctly classified 73.12% of patients. Variables excluded from the third final step were BBS, 10MWT, and ATT scores at baseline (see Table 7 for details).

**Table 7:** Binary logistic regression model to test best predictors of the RBMT-3–GMI change after rehabilitation.

		**β**	**SE**	**Wald**	***p*-value**	**Odds ratio (*B*)**
Step 1	2MWT T0	0.013	0.005	6.582	**0.010**	1.013
	Constant	−1.747	0.576	9.187	0.002	0.174
Step 2	2MWTT0	0.018	0.006	9.932	**0.002**	1.019
	RBMT-3–GMI T0	−0.046	0.015	8.844	**0.003**	0.955
	Constant	1.216	1.127	1.164	0.281	3.374
Step 3	2MWT T0	0.017	0.006	8.400	**0.004**	1.017
	AVS T0	0.648	0.268	5.859	**0.015**	1.911
	RBMT-3–GMI T0	−0.059	0.017	11.237	**0.001**	0.943
	Constant	0.325	1.294	0.063	0.802	1.384

*2MWT, 2**-**Minute Walk Test; AVS, visuospatial abilities; RBMT-3–GMI, Global Memory Index; SE, standard error. p < 0.05 are reported in bold*.

**Figure 1:**
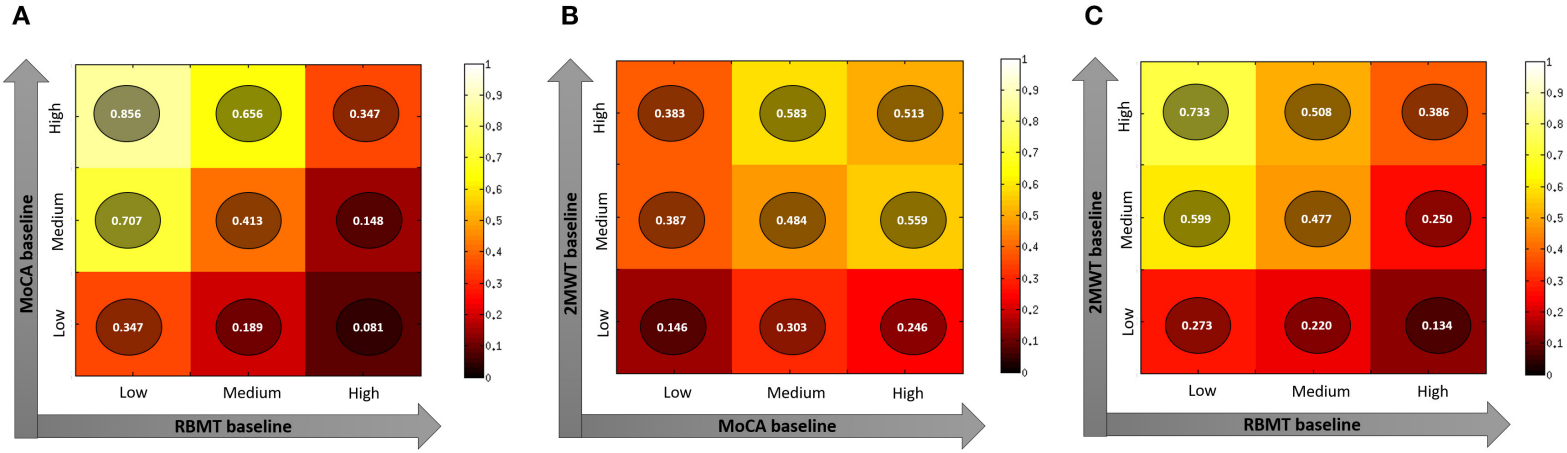
Plots representing predicted probability of different combination of best predictors (Panel **A**: MoCa and RBMT; Panel **B**: 2MWT and MoCA; Panel **C**: 2MWT and RBMT) of the RBMT-3–GMI change after rehabilitation. Panel **(A)** MoCA value at baseline and RBMT value at baseline; Panel **(B)** 2MWT value at baseline and MoCA value at baseline; Panel **(C)** 2MWT value at baseline and RBMT value at baseline. 2MWT, 2-Minute Walk Test; MoCA, Montreal Cognitive Assessment; RBMT-3–GMI, Rivermead Behavioral Memory Test-3–Global Memory Index.

Finally, when considering three-tile ranks of significant baseline predictors (RBMT-3–GMI, MoCA, and 2MWT scores–Figure 1), the ideal candidate for the HEAD treatment in the clinical setting was a person with higher residual cognitive functioning (predicted probability of success: 0.856, Figure 1, panel A) or functional mobility (predicted probability of success: 0.733, Figure 1, panel C). Moreover, an ideal candidate is a person with a higher functional mobility with a moderate level of cognitive decline (predicted probability of success: 0.583, Figure 1, panel B).

## Discussion

In the present study, we aimed to identify predictors of the HEAD treatment success considering changes in RBMT-3, an ecological measure of functional memory, and to characterize the profile of the ideal candidate for HEAD treatment.

Overall, in line with our previous reports (24), the present work clearly showed that a relatively large number of patients benefited from the HEAD treatment in the clinical setting, with a stable condition or a significant improvement, above the MCID, in all cognitive, motor, and affective domains. It is worth noting that participants were people with chronic diseases who tend to have a stable or worsening disease course over time.

Our findings on predictors of treatment success highlighted the role both of cognitive and motor abilities on the improvement in functional memory. In more detail, when delineating the profile of the ideal candidate for the HEAD treatment in clinic, we found that the prototypical patient who can report beneficial effects with a high probability is a person with more preserved general cognitive functioning and/or higher functional mobility.

Patients with higher MoCA score at baseline are not only patients with more global residual cognitive abilities, but also people with greater cognitive control. In fact, the MoCA test is a screening test highly sensitive to executive functioning, attention and visuospatial abilities (46). Individuals with higher MoCA scores should present with higher capability in representing and maintaining information about goals to be achieved over time, such as rehabilitation goals (47, 48). On the contrary, patients with less cognitive control are likely to encounter difficulties in maintaining representations of task objectives and also in shifting attention between different stimuli in the same task or between different tasks (task-shifting). Therefore, ultimately, they are unfortunately likely to benefit less from a rehabilitative treatment. Similarly, best responders in regaining functional memory after the HEAD treatment are patients with better functional mobility. In fact, persons with higher 2MWT scores at baseline are also persons with higher aerobic capacity and endurance, which represent other relevant prerequisites to perform HEAD activities and thus to achieve rehabilitation goals.

Interestingly, we observed that a high level of residual abilities in one of the two domains (cognitive or motor functioning) was sufficient to compensate the initial decay in the other one. Especially, the best treatment responders were participants with high residual level of motor abilities and a moderate residual level of cognitive functions, especially visuospatial abilities. *Vice versa*, people with high level of residual cognitive functions but moderate motor abilities benefited from the treatment with a considerable probability of success. This cognitive–motor balance underlines the critical role of the rearrangement mechanisms of the residual resources in the pathological conditions.

Our results also shed light on the intrinsic relationship subsisting between motor and cognitive functions, as well as reported in the literature. In fact, evidence showed the beneficial effects of physical activity on cognitive functions in healthy and pathological conditions (49, 50), indicating also the motor enhancement as a protective factor against cognitive impairment. The underlying biological mechanisms comprise the increment of neurotrophin level (51), the neurogenesis (52), the vascularization and angiogenesis (53), and increased activation in the frontoparietal network and a decreased activation in the default-mode network (54).

Accordingly, high cognitive control and motor abilities allowed performing motor–cognitive dual-task activities included in the HEAD treatment, demanding a discrete level of residual motor and cognitive resources. Although the potential of dual-task training has been demonstrated in different clinical populations (55, 56), potential downsides have been noted in terms of motor and cognitive interference in people with moderate disability, such as increased episodes of falls and sway (57). Moreover, motor–cognitive interference is particularly frequent in some clinical conditions, such as MS (58).

Finally, the VR devices of HEAD rehabilitation required patients to carry out quite sophisticated movements during cognitive activities, as well as visual exploration during motor and cognitive tasks. It is well-known that VR treatments particularly engage visuospatial abilities (59). This aspect could have represented a practical limitation for people with a severe disability. In fact, although there are numerous advantages related to these innovative tools, a recent study indicated also some possible barriers (60), including the need of adaptation of the technological devices to the patient's disability and the patient's additional effort in learning how to interact with the technological system.

The fact that demographic characteristics, such as age and pathology, were not significant predictors was unexpected. The lack of significant impact of age and pathology as predictors could be related to the intrinsic nature of HEAD. This treatment was conceived and developed to ensure a good level of personalization in terms of activities' contents, types, and difficulty level in clinic and at home (i.e. telerehabilitation). This aspect of the treatment allowed adapting the program session-to-session according to the patient's profile and performance. Especially, the personalization of the treatment was designed also on the basis of the pathology, in terms of the activities most effective for the specific clinical conditions (such as “finger-tapping” task for PD patients), and age, in terms of VR contents to be selected for the task (e.g., more or less up-to-date video clips). The selection of the activity's multimedia content could also be tailored to engage the patients by considering motivational aspects. Accordingly, positive outcomes related to VR rehabilitation have been reported, giving the opportunity to set numerous parameters through technological systems (61) in favor of the personalization of rehabilitation.

This work is not without limitations. We considered three clinical populations, and therefore the selection of the outcome measures for this study purposely excluded tests and scales mainly sensible to particular characteristics of a single clinical population (such as Box and Block Test for stroke). Moreover, our results are only related to cognitive outcomes and to the application of the VR in the clinical context. Future studies could adopt this approach and apply it to compare different rehabilitation settings (clinic vs. home), for detecting the impact of VR on different outcomes (i.e., quality of life, gait, affectivity…) and different cognitive domains.

To conclude, our findings will support clinical decision by identifying patients who can be targeted with high probability of VR rehabilitation success on ecological memory functioning. The ideal candidate for HEAD treatment is a person with residual capabilities on motor or cognitive domain, confirming the considerable importance of a prompt multidimensional rehabilitation and the intrinsic relationship subsisting between motor and cognitive functions. Especially, when a domain is impaired, the residual capability allows a compensative mechanism to help facilitate a successful outcome of the rehabilitation process, confirming the beneficial effects of physical activity on cognitive functions and *vice versa*.

## Data Availability Statement

The raw data supporting the conclusions of this article will be made available by the authors, without undue reservation.

## Ethics Statement

The studies involving human participants were reviewed and approved by Ethics Committees of IRCCS Don Gnocchi Foundation, the province of Lecco, Como and Sondrio Ethics Committees, and Città della Salute e della scienza of Turin Ethics Committees. The patients/participants provided their written informed consent to participate in this study.

## Author Contributions

FB, FM, and MS conceived the study. CG, JJ, and PG carried out the study. CC, CP, and SC collected data. SD, SI, and FB performed statistical analysis and interpreted results. SD, FB, and SI wrote the first draft of manuscript. All authors reviewed and approved the final manuscript.

## Conflict of Interest

The authors declare that the research was conducted in the absence of any commercial or financial relationships that could be construed as a potential conflict of interest.
